# Investigating the need for antibiotic supplementation to the extender used for semen cryopreservation in collared peccaries

**DOI:** 10.3389/fvets.2022.954921

**Published:** 2022-09-02

**Authors:** Samara S. J. Moreira, Caio S. Santos, Thibério S. Castelo, Luana G. P. Bezerra, Érica C. G. Praxedes, Tayná M. Matos, João B. F. Souza-Junior, Francisco M. C. Feijó, Pierre Comizzoli, Alexandre R. Silva

**Affiliations:** ^1^Laboratory of Animal Germplasm Conservation, Federal Rural University of Semi-Arid (UFERSA), Mossoró, RN, Brazil; ^2^Laboratory of Veterinary Microbiology, UFERSA, Mossoró, RN, Brazil; ^3^Smithsonian National Zoo and Conservation Biology Institute, Washington, DC, United States

**Keywords:** wildlife, biobank, antibiotics, semen extender, bacterial load, collared peccary

## Abstract

The objective was to investigate the effects of semen freezing extender supplementation with antibiotics on bacterial load of semen samples, sperm functional and morphological metrics in the collared peccary. Fresh ejaculates from 10 males were extended in Tris-egg yolk-glycerol supplemented or not (control) with gentamicin (70 μg/mL) streptomycin-penicillin (SP; 1 mg/mL−1000 IU/mL) or and cryopreserved in liquid nitrogen. Bacterial load, sperm motility patterns, morphology, membrane functionality and integrity, mitochondrial activity, chromatin integrity and sperm-binding ability were evaluated in fresh and frozen-thawed samples. Regardless of the use of antibiotics, the sole cryopreservation provoked a significant decrease (*P* < 0.05) in bacterial load compared to fresh samples (from average values > 1 x 10^6^ CFU/mL to <0.4 × 10^6^ CFU/mL). Post-thawing sperm kinetic parameters were not affected by the absence or presence of different antibiotics, except for beat cross frequency that was significantly (*P* < 0.05) impaired by SP supplementation compared to the group without antibiotics. After thawing, sperm morphology, membrane functionality and integrity, and mitochondrial activity were also not affected by the presence or absence of antibiotics; however, a significant decrease was observed in the group without antibiotics (*P* < 0.05) in comparison to fresh samples. Regarding sperm-binding ability, there were no differences among the different groups. While collared peccary semen could be efficiently cryopreserved in the absence of antibiotics in the extender, the use of both gentamicin or the streptomycin-penicillin combination is recommended as effective antibiotic supplementation for a further control of bacterial loads without affecting sperm parameters.

## Introduction

The collared peccary (*Pecari tajacu*) is a member of the Tayassuidae family that positively impacts Latin American ecosystems by acting as a seed disperser and prey for large carnivores. Despite being a species globally classified as stable, populations have been declining in various biomes like the Atlantic Forest (1). Thus, several efforts have been made to improve management in captivity and conservation, especially through the development of protocols for semen preservation and artificial insemination (2). Given the critical role of microbiomes on reproductive functions, the presence of *Staphylococcus spp*. and *Corynebacterium spp*. in the foreskin and semen of peccaries was recently investigated, with the proliferation of the latter bacterium significantly associated with damage to sperm membrane integrity and some kinetic parameters (3).

Negative effects of bacterial contamination in some sperm morphological (4) and functional (5) parameters have been reported. Thus, the use of antibiotics in semen extenders during freezing can contribute to the preservation of the quality and safety of male germplasm banks, especially because most of the microorganisms resulting from the semen processing can survive liquid nitrogen temperatures (−196°C) (6). On the other hand, the indiscriminate use of antibiotics can induce bacterial resistance (7). Besides, some antibiotics have been reported to negatively affect sperm quality in different species in a dose-dependent manner (8). Therefore, the possibility of avoiding antibiotic use in extenders for semen cryopreservation have been discussed, mainly because the cryopreservation process itself also causes a reduction in the bacterial load (9).

Thus, we aimed to investigate the need for antibiotic supplementation to the extender for collared peccary semen cryopreservation, by comparing the effect of samples without antibiotics with those supplemented with different antibiotics on the bacterial load, sperm functional and morphological metrics, and the sperm-binding ability.

## Materials and methods

The ethics committee of the Federal Rural University of the Semi-Arid (UFERSA) approved the experimental protocols and procedures for the care of animals used in the experiment (n°. 05/2020). The study was authorized by the Chico Mendes Institute for Biodiversity (Opinion n° No. 37329/3). All chemicals used were purchased from Sigma Chemical Co. (St. Louis, MO, USA), unless otherwise specified.

Ten sexually mature collared peccaries of 40 months of age on average were used in the study. They were grouped in a maximum of three animals that were conditioned in paddocks (20 m × 3 m) containing a covered area (3 m × 3 m) under a natural photoperiod of 12 h, at the Center for Wild Animals Multiplication located on the UFERSA campus, Mossoró, Brazil (5°10'S-37°10'W; average temperature range, 27–29°C). During the study period, the animals were fed an isocaloric (3,300 kcal/kg) and isoprotein (14% protein) diet, supplemented with tropical fruits, in addition to water *ad libitum* (2).

The animals were fasted for 12 h before the procedures and first restrained with a net, followed by an anesthetic protocol with propofol (Propovan^®^, Cristália, Fortaleza, Brazil) in bolus (5 mg/kg) intravenously. Throughout the procedure, the animals' heart and respiratory rates were monitored (10). The animals were placed in lateral recumbency and submitted to semen collection using the electroejaculation protocol previously established for the species (11) consisting of a portable device (Autojac^®^, Neovet, Campinas, SP, Brazil) connected to a 12-V source. The stimulatory cycle comprised 10 stimuli in each voltage, starting from 5 V, followed by a voltage increase in steps from 1–12 V. Each electrical stimulus lasted 3 s, with intermittentbreaks of 2 s. The stimuli cycle was maintained for a 10-min duration from the beginning of the procedure. The electroejaculator probe was 15 1.3 cm, and it was inserted 12 cm into the rectum. Ejaculates were collected in plastic tubes and individually evaluated and processed.

For the microbiological analysis, an aliquot of 100 μL of each semen sample was inoculated in 900 μL of sterile 0.85% saline, obtaining a dilution of 10–1, followed by a serial dilution up to 10–5. Aliquots of 100 μL of each dilution were sown, with the aid of a Drigalski loop, on the surface of Petri dishes containing Plate Count Agar (Hi Media, Mumbai, India), all in duplicate and incubated in a bacteriological incubator (Fanem LTDA, São Paulo, Brazil) at 37°C for 24–48 h. Colonies were then counted on each plate and the number of microorganisms was expressed in Colony Forming Unit–CFU/mL multiplied by the inverse of each dilution (12).

Ejaculates were evaluated for color and appearance. The volume was measured using micropipettes, and the pH was determined using pH indicator strips (Neutralit^®^, Merck, Bucharest, Romania). Sperm concentration (millions of sperm/mL) was estimated in a Neubauer counting chamber (13).

Sperm kinetic patterns were analyzed using computerized semen analysis (IVOS 7.4 G; Hamilton-Thorne Research, Beverly, MA, USA), using settings previously established for peccaries (13). The settings of the instrument included temperature 37°C; 60 frames/s; minimum contrast, 45; straightness threshold, 30%; low-velocity average pathway (VAP) cutoff, 10 m/s; and medium VAPcutoff, 30 m/s. Five independent and non-consecutive microscopic fields were randomly selected and evaluated using scanning procedures. Values for the following parameters were analyzed: number of cells counted, total motility (%), velocity average pathway (VAP; mm/s), straight-line velocity (VSL: mm/s), curvilinear velocity (VCL: mm/s), amplitude lateral head (ALH; mm), beat cross frequency (BCF; Hz), straightness (STR; %) and linearity (LIN; %), as well as the sperm subpopulations: fast, medium, slow and static. For a reliable assessment of sperm motility patterns, the Edit Tracks option of the IVOS 7.4 G system was used to exclude the debris derived from the extenders. A further dilution in salt solution (1:1) was conducted only if necessary (13).

Morphological analysis was performed using semen smears stained with Bengal Rose and evaluated under light microscopy (×1000; 200 cells/slide) (13). The functionality of the sperm membrane was analyzed through the osmotic response of sperm to the hypo-osmotic test with distilled water (0 mOsm/L), evaluated under light microscopy (x400; 200 cells/slide) (13). Chromatin integrity was assessed using a smear stained with toluidine blue dye [0.025% dye in McIlvaine buffer (sodium citrate: phosphate pH ¼ 4.0)] and evaluated under light microscopy (x1000; 500 cells; slide). Cells stained slightly blue were classified as normal (negative) and those stained from violet to dark blue were considered to have altered chromatin (positive) (14).

Sperm membrane integrity and mitochondrial activity were assessed using a combination of fluorescent solutions: 3 μL of Hoechst 342 (H342; Sigma-Aldrich, St Louis, MO, USA) (998.4 μL of DPBS + 1.6 μL of stock solution: 25 μg/mL), 5 μL of CMXRos (Mito Tracker red^®^, Molecular Probes, M-7512) (1 mL of TRIS + 0.1 μL of stock solution: 50 μg / 94 μL) and 2 μL of IP (Propidium Iodide, Sigma-Aldrich, Co., St Louis, MO, USA) (980 μl DPBS + 20 μl stock solution: 25 μg/ml). A total of 200 cells were evaluated under an epifluorescence microscope (Episcopic Fluorescent attachment EFA Halogen Lamp Set. Leica. Kista, Sweden), whose spermatozoa with a blue head (H-342) were judged to contain an intact membrane and those with a full head or partially red-labeled (PI) were considered to contain a non-intact membrane, and a red-labeled midpiece were considered having a mitochondrial function (12).

The binding ability of spermatozoa was investigated using the hen egg perivitelline membrane binding assay, as previously validated for peccaries (15). Briefly, egg yolk membranes from fresh and non-fertile chicken eggs were washed in saline solution at 37°C and submitted to 1 cm^2^ cuts, with two membranes for each treatment. Together, the sperm samples (1: 1) were diluted in an incubation medium solution (114 mM NaCl; 3.1 mM KCl; 0.4 mM NaH_2_PO_4_; 10 mM calcium lactate; 25 mM NaHCO_3_; 10 μg/mL phenol red; 1.4 mM caffeine; 2.0 mM CaCl_2_.2H_2_O; 0.5 mM MgCl_2_; 10 mM Hepes; 6 mg/mL BSA; 5.5 mM glucose; 0.45 mM sodium pyruvate; 40 μg/mL gentamicin; pH 7.47.8), and subsequently centrifuged at 700 xg for 10 min. The pellet was resuspended to obtain 1 x 10^6^ sperm/mL that was incubated in a 4-well plate with a membrane fragment at 38.5°C for 20 min in a water bath. After incubation, each membrane was washed in 100 μL drops of incubation medium for the removal of non-binding sperm, and subsequently kept in Hoechst 33250 for 15 min. Finally, the membranes were evaluated for the number of ligating sperm, evaluating six distinct and random fields using epifluorescence microscopy (Episcopic Fluorescent attachment EFA Halogen Lamp Set. Leica. Kista, Sweden).

For freezing, the ejaculates were diluted in Tris plus glycerol (3%) and egg yolk (20%), and separated into three aliquots that were kept without antibiotics (Control group) or supplemented with gentamicin (70 μg/mL) or streptomycin/penicillin (1 mg/mL/1000 IU/mL), as previously reported for peccary semen refrigeration (12). Final dilution resulted in a 100 x 10^6^ sperm/mL concentration. The samples were refrigerated at 15°C for 40 min in isothermal boxes and stabilized at 5°C for another 30 min in a biological incubator (Quimis, Diadema, SP, Brazil). Then, they were filled in 0.25 mL plastic that were placed in contact with the nitrogen vapor (5 cm) for 5 min and finally stored in a cryobiological container at −196° C. After 1 week, the samples were thawed in a water bath at 37°C for 1 min (13) and evaluated for microbiological load and sperm metrics as described for fresh samples.

A total of 10 samples obtained from 10 animals (one sample per animal) were used in the experiment. Each individual sample was divided into three aliquots, which were allocated to each of the treatments tested. Data obtained were expressed as mean ± standard error of 10 replicates. Normality of residual was verified by the Shapiro-Wilk test and homogeneity of variance by Levene's test. The data of total motility, medium subpopulation, and membrane functionality and integrity were transformed into arcsine to attend to the parametric assumptions. Initially, fresh semen was considered as **one** of the treatments and Dunnett's test was applied to compare it with the other treatments. Subsequently, a one-way ANOVA, followed by Tukey's *post hoc* test to evaluate differences among treatments (thawed) were performed. For all analyses, Statistical Analysis Software, version 8.0 (SAS Institute Inc., Cary, NC, USA) was used and in all pairwise comparisons, a *P* < 0.05 was considered.

## Results

**Figure 1:**
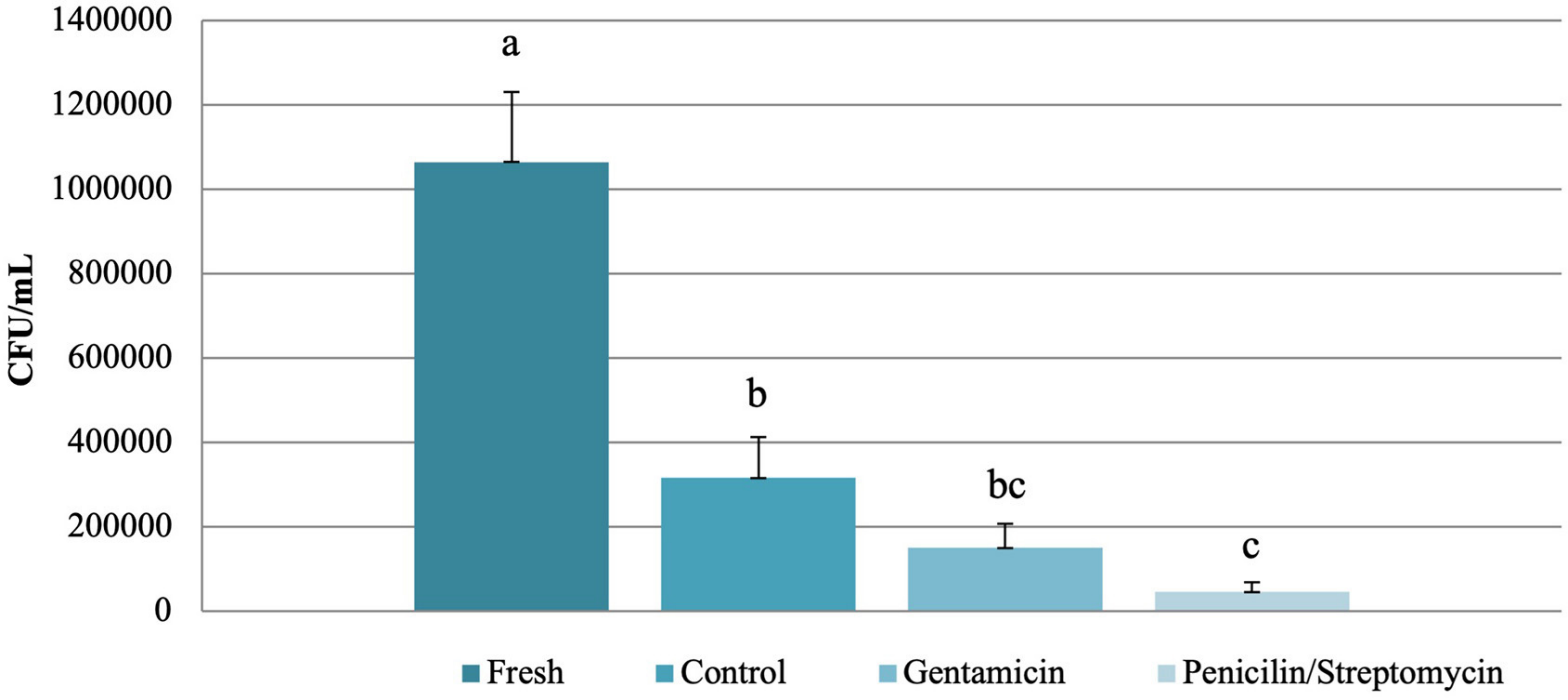
Mean (± SEM) values for bacterial load (CFU/mL) in fresh and frozen-thawed semen from collared peccaries (*n* = 10) supplemented of not with different antibiotics (gentamicin and streptomycin-penicillin combination). a–c Lowercase letters indicate significant differences for fresh and treatment groups (Dunnett's test; *P* < 0.05).

Cryopreservation alone (values ranging from 0.04 to 1 x 10^6^ to CFU/mL) already caused a significant decrease (*P* < 0.05) in bacterial load compared to fresh samples (values ranging from 0.4 to 21.3 x 10^6^ CFU/mL). The most significant decrease (*P* < 0.05) of bacterial load was observed after the use of the streptomycin-penicillin combination (values ranging from 0 to 0.2 x 10^6^ CFU/mL), while values from the gentamicin group (values ranging from 0.01 to 0.5 x 10^6^ CFU/mL) did not differ from either the streptomycin-penicillin or the non-antibiotic control group (Figure 1).

Ejaculates presented a milky appearance, with a whitish color, and pH 7.5 ± 0.2. The average volume was 5.0 ± 1.2 mL, with average sperm concentration of 461.0 ± 59.2 × 10^6^ sperm/mL. The mean number of total motile spermatozoa was 95.3 ± 0.8%, with 80.0 ± 4.3% morphologically normal cells, being 69.4 ± 8.6% with functional membrane, 80.9 ± 2.2% with intact membrane, 79.3 ± 2.8% with mitochondrial activity, and 99.4 ± 0.2% with normal condensed chromatin.

After thawing, there was a significant decrease (*P* < 0.05) on most of the sperm kinetic parameters (Table 1) in comparison to fresh samples. However, post-thawing values for VCL, ALH, straightness, medium and slow populations in all the experimental groups were similar to those observed for fresh semen (*P* < 0.05). Furthermore, there were no differences among experimental groups for these kinetic parameters (*P* > 0.05). Regarding Beat Cross Frequency (Table 1), values obtained for the control group without antibiotics were the only similar to those observed for fresh samples (*P* > 0.05), while the group containing streptomycin-penicillin significantly impaired this parameter when compared to other treatments (*P* < 0.05).

**Table 1:** Mean (± SEM) values for kinetic motility patterns in fresh and frozen-thawed semen from collared peccary (*n* = 10) supplemented or not with different antibiotics (gentamicin and streptomycin-penicillin combination).

	**Fresh**	**Frozen-Thawed**		
**Sperm kinetic motility patterns**		**Control**	**Gentamicin**	**Streptomycin-penicillin**
Total motility (%)	95.3 ± 0.8^A^	34.1 ± 3.7^Ba^	37.2 ± 6.1^Ba^	32.6 ± 4.0^Ba^
Progressive motility (%)	72.1 ± 3.5^A^	20.2 ± 2.4^Ba^	23.2 ± 4.6^Ba^	20.0 ± 2.8^Ba^
Velocity average pathway (mm/s)	69.2 ± 4.8^A^	46.1 ± 2.7^Ba^	47.7 ± 2.8^Ba^	45.8 ± 2.5^Ba^
Velocity straight line (mm/s)	56.8 ± 4.4^A^	35.7 ± 3.1^Ba^	36.0 ± 3.4^Ba^	33.2 ± 3.0^Ba^
Velocity curvilinear (mm/s)	119.4 ± 8.0^A^	100.6 ± 5.5^Aa^	100.2 ± 4.4^Aa^	99.7 ± 5.0^Aa^
Amplitude lateral head (mm)	5.3 ± 0.3^A^	5.4 ± 0.2^Aa^	5.6 ± 0.1^Aa^	5.7 ± 0.2^Aa^
Beat cross frequency (Hz)	37.2 ± 0.6^A^	35.6 ± 0.9^Aa^	34.1 ± 0.5^Bab^	31.7 ± 1.0^Bb^
Straightness (%)	77.5 ± 2.0^A^	72.7 ± 2.4^Aa^	70.9 ± 3.5^Aa^	69.0 ± 2.2^Aa^
Linearity (%)	47.0 ± 2.4^A^	34.4 ± 1.5^Ba^	34.8 ± 2.3^Ba^	33.3 ± 1.5^Ba^
**Sperm subpopulations**
Rapid (%)	81.0 ± 3.6^A^	23.5 ± 3.0^Ba^	27.7 ± 4.9^Ba^	23.4 ± 3.0^Ba^
Medium (%)	14.4 ± 3.0^A^	10.6 ± 1.8^Aa^	9.4 ± 1.4^Aa^	9.1 ± 1.6^Aa^
Slow (%)	2.0 ± 0.2^A^	2.7 ± 0.7^Aa^	2.6 ± 0.4^Aa^	2.4 ± 0.6^Aa^
Static (%)	3.0 ± 0.6^A^	63.1 ± 4.2^Ba^	60.3 ± 6.4^Ba^	65.0 ± 4.1^Ba^

^AB^Superscript capital letters indicate a significant difference between fresh and thawed groups (Dunnett's test; P < 0.05).

^ab^Superscript lower case letters indicate significant difference between experimental groups (Tukey's test; P < 0.05).

Regarding sperm morphology (Table 2), similar values were observed among fresh samples and all the post-thawing experimental groups (*P* > 0.05). For the analysis of chromatin integrity (Table 2), groups containing antibiotics provided similar values as those observed for fresh samples (*P* > 0.05); however, a significant decrease on this parameter was observed for the samples cryopreserved without antibiotics (*P* < 0.05). For membrane functionality and integrity, and mitochondrial activity (Table 2), there was a significant decrease (*P* < 0.05) after thawing in comparison to fresh semen (*P* < 0.05), but no significant differences were observed among treatment groups (*P* > 0.05).

**Table 2:** Mean (± SE) values for sperm normal morphology, membrane functionality and integrity, mitochondrial activity and chromatin integrity in fresh and frozen-thawed semen from collared peccaries (*n* = 10) supplemented or not with different antibiotics (gentamicin and streptomycin-penicillin combination).

	**Fresh**	**Frozen-Thawed**		
**Sperm Parameters**		**Control**	**Gentamicin**	**Streptomycin-Penicillin**
Normal morphology (%)	80.0 ± 4.3^A^	79.4 ± 2.2^Aa^	75.1 ± 3.5^Aa^	79.1 ± 3.3^Aa^
Membrane functionality (%)	69.4 ± 8.62^A^	50.0 ± 3.6^Ba^	59.4 ± 6.1^Ba^	50.5 ± 4.3^Ba^
Chromatin integrity (%)	99.4 ± 0.2^A^	97.5 ± 0.6^Ba^	98.5 ± 0.5^ABa^	98.4 ± 0.6^ABa^
Membrane integrity (%)	80.9 ± 2.2^A^	37.6 ± 5.4^Ba^	40.1 ± 5.8^Ba^	31.3 ±2.5^Ba^
Mitochondrial activity (%)	79.3 ± 2.8^A^	31.3 ± 5.4^Ba^	34.9 ± 5.9^Ba^	30.6 ± 2.8^Ba^

^AB^Superscript capital letters indicate a significant difference between fresh and thawed groups (Dunnett's test; P < 0.05).

^abc^Superscript lowercase letters indicate a significant difference between the experimental groups (Tukey's test; P < 0.05).

Regarding sperm-binding assay (Table 3), values of 212.0 ± 22.6 sperm bound to perivitelline membranes were observed for fresh samples, but the cryopreservation process caused a significant decrease (*P* < 0.05) of ~50% in the values found for all experimental groups, regardless of the use of antibiotics.

**Table 3:** Mean (± SE) values and range (Min—Max) for number of sperm bound to the perivitelline membrane of hen egg yolk in fresh and frozen-thawed semen from collared peccaries (*n* = 10) supplemented or not with different antibiotics (gentamicin and streptomycin-penicillin combination).

	**Fresh**	**Frozen-Thawed** ^*^		
		**Control**	**Gentamicin**	**Penicillin/Streptomycin**
Number of bound sperm	212.0 ± 22.6^A^	105.0 ± 16.1^B^	100.7 ± 8.1^B^	95.8 ± 13.4^B^
Min	99.7	34.5	47.2	47
Max	351.2	185.5	148.3	196.7

^AB^Superscript capital letters indicate significant difference between fresh and frozen-thawed groups (Dunnett's test; P < 0.05).

* There were no differences among post-thawing experimental groups (Tukey's test; P > 0.05).

## Discussion

While the sole cryopreservation process can cause a significant reduction in semen sample's bacterial loads, the study demonstrated the possibility of supplementing semen extender with different antibiotics for further control of bacterial load, without provoking extensive damage on sperm quality during the cryopreservation process.

Regarding bacterial contamination in peccary fresh ejaculates, we found values >1 x 10^6^ CFU/mL, which are into the normal range previously described for the species as 0.04 to 2.2 x 10^6^ CFU/mL (3). It is difficult to compare these findings with those reported for other species, as even in boars there is no consensus regarding the number of bacteria found in fresh ejaculates, which can vary from just 0.08 10^6^ CFU/mL (16) to 370 x 10^6^ CFU/mL (17). In fact, semen collection in farm animals is not a sterile procedure, with some bacterial flora contaminating the semen (16), but the adoption of adequate sanitary practices can help to reduce sample contamination.

In general, the combination of streptomycin-penicillin, as well as gentamicin, are among the antibiotics most commonly used in the composition of extenders for the semen of farm animals (8), which is why these were the antibiotics chosen to be used in the present study. The use of antibiotics during peccary semen freezing allowed an effective control of bacterial load. Both streptomycin and gentamicin are aminoglycosides that acts in the protein synthesis of gram-negative bacterial cells (18, 19). In the other hand, the penicillin is a β lactam that acts by inhibiting the synthesis of cell wall of gram-positive bacteria (19). Even if this is the first time that these antibiotics were used of the cryopreservation of peccary sperm, they were previously proven to provide efficient control of bacterial load during peccary semen chilling for 36 h. At this point, the streptomycin-penicillin combination was able to reduce the bacterial load to zero in various samples (12).

As demonstrated by the present study, freezing conditions alone could be maintain contamination at acceptable levels in peccary semen as previously observed for ram (9). It has already been shown that bacteriostasis can be achieved during storage of swine semen even at a temperature of 5°C in the absence of antibiotics (20). Furthermore, in domestic swine, it is assumed that a complete elimination of microorganisms in semen samples cannot be justified, given the high exposure of the female genital tract to commensal bacteria in ejaculates during natural mating, which could play a role in important physiological immunogenic role (20). Therefore, the adoption of strict hygiene measures should be focused on avoiding the exposure of animals to pathogenic microorganisms that could then be transmitted *via* semen samples.

Besides controlling bacterial load, the treatment containing no antibiotics was able to provide an effective preservation of all sperm metrics, except the chromatin integrity of frozen-thawed peccary sperm. As reported for human sperm, bacterial contamination can directly alter the sperm function, increasing the phosphatidylserine translocation and the apoptosis activation, which could be related to DNA condensation and fragmentation (5). In collared peccaries, however, the aforementioned deleterious effect on chromatin integrity does not seem to be reflected on fertility, since a sperm binding capacity similar to the groups containing antibiotics was observed. Besides it, no differences were observed between the groups containing or not containing antibiotics in relation to the rates obtained in the sperm binding test, despite differences relative to BCF especially with the inclusion of SP to the extender. Furthermore, there were no differences between groups with or without antibiotics regarding post-thawing sperm morphology, membrane functionality and integrity, and mitochondrial activity.

The discussion concerning the need to control microorganisms present in semen is still quite controversial, especially for wild animal species. If, on one hand, the microbiome of wild animals is still unknown, and its impact on the physiology of these species remains to be elucidated (21), on the other hand, there is a possibility that biological samples collected in the field may transmit unknown microorganisms to captive populations or even for men (22). In this context, our results provide novel information related to the possibility of freezing collared peccary semen samples without the presence of antibiotics. It is worth mentioning that the animals used in the present study have been bred in captivity for several generations, and the description of their reproductive microbiome had already been previously performed (3). Besides, semen collection procedures were carried out taking all necessary sanitary precautions to reduce sample contamination. Under field conditions, however, it is not always possible to carry out the collection in an aseptic way, and the use of antibiotics may be advised for this purpose, especially if samples are destined to *in vitro* fertilization trials (23).

In conclusion, it is noteworthy that the cryopreservation process alone can control bacterial load, without promoting effective damage to collared peccary semen samples. However, if necessary, the antibiotic supplementation, both gentamicin and the streptomycin-penicillin combination are indicated for the extender used in the cryopreservation of collared peccary semen. These are important findings to be considered when using germplasm from populations kept in captivity or from those living in the wild to create safe biobanks and also assisted reproductive technologies (ART) or artificial insemination (AI).

## Data availability statement

The raw data supporting the conclusions of this article will be made available by the authors, without undue reservation.

## Ethics statement

The animal study was reviewed and approved by the Ethics Committee of the Federal Rural University of the Semi-Arid (UFERSA) approved the experimental protocols and procedures for the care of animals used in the experiment (n°. 05/2020). The study was authorized by the Chico Mendes Institute for Biodiversity (Opinion n° No. 37329/3).

## Author contributions

All authors equally contributed for manuscript conceptualization, methodology, data analysis, and writing and approved the submitted version.

## Funding

This study was supported by Coordenação de Aperfeiçoamento de Pessoal de Nível Superior—Brasil (CAPES, Financial Code 001). AS is a receipt of grants from Brazilian National Council for Scientific and Technological Development (CNPq).

## Conflict of interest

The authors declare that the research was conducted in the absence of any commercial or financial relationships that could be construed as a potential conflict of interest.

## Publisher's note

All claims expressed in this article are solely those of the authors and do not necessarily represent those of their affiliated organizations, or those of the publisher, the editors and the reviewers. Any product that may be evaluated in this article, or claim that may be made by its manufacturer, is not guaranteed or endorsed by the publisher.
